# Analysis of the Conduction Mechanism and Copper Vacancy Density in p-type Cu_2_O Thin Films

**DOI:** 10.1038/s41598-017-05893-x

**Published:** 2017-07-18

**Authors:** Sanggil Han, Andrew J. Flewitt

**Affiliations:** Electrical Engineering Division, Department of Engineering, University of Cambridge, Cambridge, CB3 0FA United Kingdom

## Abstract

A quantitative and analytical investigation on the conduction mechanism in p-type cuprous oxide (Cu_2_O) thin films is performed based on analysis of the relative dominance of trap-limited and grain-boundary-limited conduction. It is found that carrier transport in as-deposited Cu_2_O is governed by grain-boundary-limited conduction (GLC), while after high-temperature annealing, GLC becomes insignificant and trap-limited conduction (TLC) dominates. This suggests that the very low Hall mobility of as-deposited Cu_2_O is due to significant GLC, and the Hall mobility enhancement by high-temperature annealing is determined by TLC. Evaluation of the grain size and the energy barrier height at the grain boundary shows an increase in the grain size and a considerable decrease in the energy barrier height after high-temperature annealing, which is considered to be the cause of the significant reduction in the GLC effect. Additionally, the density of copper vacancies was extracted; this quantitatively shows that an increase in annealing temperature leads to a reduction in copper vacancies.

## Introduction

Cuprous oxide (Cu_2_O) is a promising candidate as an active layer for p-type oxide semiconductor thin film transistors (TFTs). This is because hole-producing copper vacancies (*V*_*Cu*_) are easily created due to their low formation energy, and effectively provide holes in Cu_2_O because of the small acceptor ionisation energy (i.e. shallow acceptor level), which leads to intrinsically stable p-type conductivity^1^. Furthermore, comparable energy levels of Cu 3*d* and O 2*p* orbitals introduce considerable covalency into the ionic metal-oxide material system^1–3^. This not only reduces the localization of holes around negatively charged oxygen ions, but also disperses the valence band^1^, which theoretically enables it to have a Hall mobility of ~270 cm^2^/V · s at room temperature^4^. However, the presence of valence band tail states and potential barriers at grain boundaries leads to deposited thin films of Cu_2_O having a significantly lower hole mobility compared with this theoretical limit.

To be specific, the nanocrystralline structure of thin Cu_2_O films suggests the presence of potential energy barriers at grain boundaries. The effect of the potential barriers such as grain boundary scattering (i.e. grain-boundary-limited conduction, GLC) impedes hole transport^5, 6^. Furthermore, if the width of the tail states in nanocrystalline materials is similar to or larger than the thermal energy at room temperature, the tail states also have a strong effect on carrier transport since a large number of thermally excited carriers are trapped at the band tail states^7^. Whilst the conduction band minimum (CBM) in Cu_2_O is formed from spherical overlapping Cu 4*s* orbitals, the valence band maximum (VBM) is due to non-spherical Cu 3*d* orbitals which have spatial directivity, and thus they are sensitive to bonding angle disorder^8^. As in disordered silicon where the VBM is mainly composed of non-spherical *p* orbitals^9^, this creates a broad distribution of localised tail states near the VBM of Cu_2_O films. The Urbach energy (*E*_*u*_) is a parameter reflecting the width of the tail states and thin Cu_2_O films show an *E*_*u*_ larger than the thermal energy^8, 10^. Thus, multiple carrier trapping and thermal release of holes in tail states (i.e. trap-limited conduction, TLC) also degrades transport in Cu_2_O^11–13^.

Post-deposition annealing of Cu_2_O has been reported as one of the ways to enhance the performance of Cu_2_O TFTs^14, 15^. In a recent report^8^, we showed that high-temperature annealing in vacuum leads to a significant improvement in the field-effect mobility and a reduction in the off-state current, mainly resulting from a film mobility (i.e. Hall mobility, *μ*_*Hall*_) enhancement and a decrease in intrinsic carrier density (i.e. free hole concentration, *p*_*free*_), respectively. The changes in *μ*_*Hall*_, *p*_*free*_ and *E*_*u*_ according to annealing temperature (*T*_*A*_) are summarized in Table 1.

**Table 1:** Summary of Parameters (*μ*_*Hall*_, *p*_*free*_ and *E*_*u*_) for Different Annealing Temperatures^8^.

Annealing Temperature [°C]	As-deposited	500	600	700
***μ***_***Hall***_ [cm^2^/V ∙ s]	0.14	3.75	7.42	28
***p***_***free***_ [cm^−3^]	1.68 × 10^16^	1.30 × 10^14^	7.34 × 10^13^	1.85 × 10^13^
***E***_***u***_ [meV]	223	166	128	78

In this paper, an analytical study on the conduction mechanism and the density of copper vacancies in Cu_2_O thin films is presented in order to allow an in-depth discussion on the change in electrical characteristics of Cu_2_O by high-temperature annealing. The effect of GLC on *μ*_*Hall*_ is quantified using a GLC coefficient (*α*_*GLC*_) which is extracted from the difference between *p*_*trap*(*Hall*)_ (the trapped hole concentration calculated from measured *μ*_*Hall*_ which includes the effects of TLC and GLC) and *p*_*trap*(*DOS*)_ (the trapped hole concentration calculated based on extracted subgap density of states including only the TLC effect). Using the extracted *α*_*GLC*_ and Matthiessen’s rule, the relative dominance of TLC and GLC is quantitatively assessed. The density of copper vacancies $({N}_{{V}_{Cu}})$ as a function of *T*_*A*_ was extracted using an equation derived from the charge neutrality condition, with consideration for ionized valence band tail states, and the formula for the ionized acceptor concentration. This work is important for an understanding of not only the dominant mobility degradation mechanism in Cu_2_O but also the main cause of the mobility improvement by post-deposition annealing.

## Results and Discussion

### *μ*_*Hall*_ definition considering TLC and GLC

If carrier mobility is affected by several conduction mechanisms and they are independent of each other, Matthiessen’s rule (i.e. ${\mu }^{-1}={\sum }_{i}{{\mu }_{i}}^{-1}$, where *μ*_*i*_ denotes a mobility limited by a single conduction mechanism) can be applied. Using this rule, the effects of TLC and GLC can be incorporated into *μ*_*Hall*_ as follows,

1 $$\frac{1}{{\mu }_{{Hall}}}=\frac{1}{{\mu }_{0}}+\frac{1}{{\mu }_{{TLC}}}+\frac{1}{{\mu }_{{GLC}}}=\frac{1}{{\mu }_{0,{TLC}}}+\frac{1}{{\mu }_{{GLC}}},$$

where *μ*_0_, *μ*_*TLC*_ and *μ*_*GLC*_ are the free carrier mobility and the mobilities limited by TLC and GLC, respectively. Here, ${\mu }_{0}^{-1}+{\mu }_{{TLC}}^{-1}$ can be expressed as ${\mu }_{0,{TLC}}^{-1}$ (i.e. ${\mu }_{0}^{-1}+{\mu }_{{TLC}}^{-1}={\mu }_{0,{TLC}}^{-1}$, where *μ*_0,*TLC*_ is the effective carrier mobility reduced by TLC). Using trap-limited conduction theory, *μ*_0,*TLC*_ can be defined by the product of *μ*_0_ and *β*_*TLC*_ which is the ratio of free carrier concentration (*p*_*free*_) to the total carrier concentration (i.e. *p*_*free*_ + *p*_*trap*_) as follows^7^,

2 $${\mu }_{0,{TLC}}={\mu }_{0}{\beta }_{{TLC}},$$

3 $${\beta }_{{TLC}}=(\frac{{p}_{{fre}e}}{{p}_{{free}}+\,{p}_{{trap}}}),$$

where *p*_*trap*_ denotes the concentration of holes trapped in the valence band tail states. Using Equations (1) and (2), *μ*_*Hall*_ is then given by

4 $${\mu }_{{Hall}}=\frac{{\mu }_{0}{\beta }_{{TLC}}{\mu }_{{GLC}}}{{\mu }_{0}{\beta }_{{TLC}}+{\mu }_{{GLC}}}={\mu }_{0}{\alpha }_{{GLC}}{\beta }_{{TLC}},$$

5 $${\alpha }_{{GLC}}=\frac{{\mu }_{{GLC}}}{{\mu }_{0}{\beta }_{{TLC}}+{\mu }_{{GLC}}}.$$

Here, *α*_*GLC*_ is the GLC coefficient quantifying the effect of GLC on *μ*_*Hall*_ (0 < *α*_*GLC*_ ≤ 1, where *α*_*GLC*_ = 1, i.e. ${\mu }_{{GLC}}\gg {\mu }_{0}{\beta }_{{TLC}}$, represents the condition when *μ*_*Hall*_ is affected by only TLC).

### Extraction of *α*_*GLC*_

The GLC effect on *μ*_*Hall*_ was first quantified by extraction of *α*_*GLC*_. The *α*_*GLC*_ can be determined based on the difference between *p*_*trap*(*Hall*)_ and *p*_*trap*(*DOS*)_. Specifically, using Equations (3) and (4), *p*_*trap*_ is given as follows,

6 $${{p}}_{{trap}}=(\frac{{\alpha }_{{GLC}}{\mu }_{0}}{{\mu }_{{Hall}}}-1){{p}}_{{free}}.$$

Here, without considering *α*_*GLC*_, i.e.

7 $${{p}}_{{trap}({Hall})}=(\frac{{\mu }_{0}}{{\mu }_{{Hall}}}-1){{p}}_{{free}},$$

**Figure 1:**
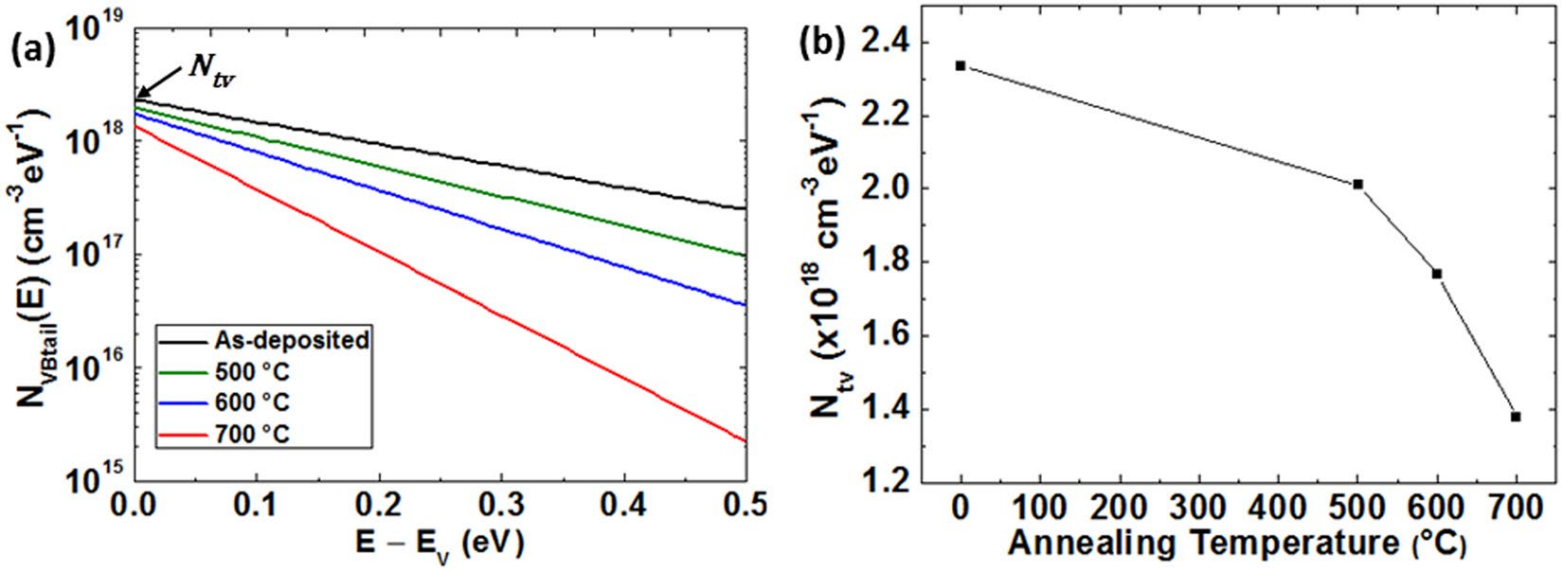
(**a**) Extracted density of tail states at the valence band (*N*_*VBtail*_(*E*)) and (**b**) tail state density at *E* = *E*_*V*_ (*N*_*tv*_) of Cu_2_O films before and after annealing.

*p*_*trap*(*Hall*)_ can be obtained using measured *μ*_*Hall*_, *p*_*free*_ and *μ*_0_ = 270 cm^2^/V · s (the theoretical limit of *μ*_*Hall*_ measured by longitudinal-optical (LO) phonon scattering at room temperature)^4, 16^. If GLC affects hole transport, *p*_*trap*(*Hall*)_ is overestimated since *α*_*GLC*_ (0 < *α*_*GLC*_ < 1), and this reflects the degradation of *μ*_0_ by GLC which is not considered in Equation (7). The *α*_*GLC*_ can be extracted from the overestimated extent of *p*_*trap*(*Hall*)_ against *p*_*trap*(*DOS*)_. The density of tail states at the valence band (*N*_*VBtail*_(*E*)) can be approximated as an exponential distribution using O’Leary’s model for the distribution of electronic states of disordered semiconductors as follows^17^,

8 $${{N}}_{{VBtail}}({E})={{N}}_{{tv}}\,\exp \,(\frac{{{E}}_{{V}}-{E}}{{{E}}_{{u}}}),$$

9 $${{N}}_{{tv}}=\frac{\sqrt{2}{{m}}_{{h}}^{\ast 3/2}}{{\pi }^{2}{\hslash }^{3}}\sqrt{\frac{{{E}}_{{u}}}{2}}\,{\rm{\exp }}\,(-\,\frac{1}{2}),$$

where *E*_*V*_, *N*_*tv*_, *E*_*u*_, *ħ* and ${{m}}_{{h}}^{\ast }$ are the valence band edge, the density of tail states at *E* = *E*_*V*_, the Urbach energy reflecting the width of the tail states, the Planck constant and the density-of-states effective mass of holes in the valence band, respectively. Since valence band states of light holes are situated at the top of the valence band^18^, the majority of holes are produced from the light hole band. For this reason, the band mass of light holes (*m*_*lh*_) can be considered as ${{m}}_{{h}}^{\ast }$, which is about 0.56 *m*_0_^18^, where *m*_0_ denotes the electron rest mass. Using Equations (8) and (9) and *E*_*u*_ in Table 1, *N*_*VBtail*_(*E*) and *N*_*tv*_ were extracted as seen in Fig. 1. The hole density trapped at the tail states (*p*_*trap*(*DOS*)_) can be calculated using Equation (8) and the Fermi-Dirac distribution function (i.e. the probability of occupation of the donor-like tail states by an electron), ${F}({E})=1/[1+{\rm{e}}xp\{({E}-{{E}}_{{F}})/{kT}\}]$, where *E*_*F*_ is the Fermi energy, as

10 $${p}_{trap(DOS)}={\int }_{{E}_{V}}^{\infty }{N}_{VBtail}(E)[1-F(E)]dE.$$

Here, we assumed that all ionised donor-like tail states filled with a hole (i.e. *p*_*trap*_) are located above *E*_*F*_ (i.e. *F*(*E*) = 0 at *E* > *E*_*F*_, *F*(*E*) = 1 at *E* < *E*_*F*_), corresponding to the condition when the tail state energy ${k}{{T}}_{{t}}\cong {{E}}_{{u}}$ (*T*_*t*_ is the characteristic temperature of the tail states) is larger than the thermal energy *kT* (see Table 1)^11, 19, 20^. This yields the solution of Equation (10) as

11 $${p}_{trap(DOS)}\approx {N}_{tv}{E}_{u}\,\exp \,(\frac{{E}_{V}-{E}_{F}}{{E}_{u}}).$$

In order to calculate *p*_*trap*(*DOS*)_, (*E*_*V*_ − *E*_*F*_) was extracted from the measured *p*_*free*_ (see Table 1) and its formula given by the Boltzmann approximation^21^,

12 $${p}_{free}={N}_{V}\,\exp \,(\frac{{E}_{V}-{E}_{F}}{kT}).$$

*N*_*V*_ is the effective density of states for free carriers in the valence band and is calculated using^21^

13 $${N}_{V}\equiv 2{(\frac{2\pi {m}_{h}^{\ast }kT}{{\hslash }^{2}})}^{3/2}.$$

**Figure 2:**
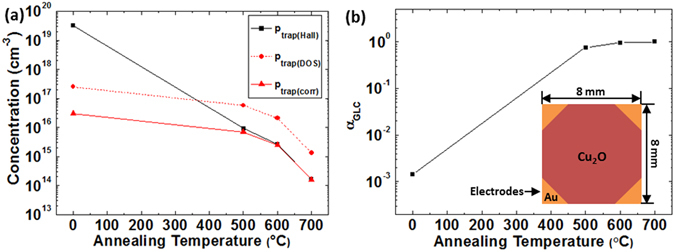
(**a**) *p*_*trap*(*Hall*)_, *p*_*trap*(*DOS*)_ and *p*_*trap*(*corr*)_ and (**b**) *α*_*GLC*_ as a function of annealing temperature. Inset shows the schematic van der pauw geometry for the Hall measurement. (**a**) shows the discrepancy between *p*_*trap*(*Hall*)_ (calculated using *μ*_*Hall*_ including the effects of TLC and GLC) and *p*_*trap*(*corr*)_ (i.e. *p*_*trap*(*DOS*)_ corrected using *p*_*trap*(*Hall*)_ (700 °C)) including only the TLC effect. In (**b**), the effect of GLC on *μ*_*Hall*_ is quantified by *α*_*GLC*_ (extracted using Equation (15) and the difference between *p*_*trap*(*Hall*)_ and *p*_*trap*(*corr*)_). This shows that *α*_*GLC*_ approaches unity as annealing temperature increases: 0.0014 (as-deposited), 0.76 (500 °C), 0.96 (600 °C), 1 (700 °C). This suggests that GLC becomes insignificant after high-temperature annealing.

For ${m}_{h}^{\ast }=0.56\,{m}_{0}$, *N*_*V*_ was calculated to be 1.05 × 10^19^ cm^−3^. Using the calculated *N*_*V*_ and Equation (12), we obtained *E*_*V*_ − *E*_*F*_ = −0.166 eV (as-deposited), −0.29 eV (500 °C), −0.31 eV (600 °C) and −0.34 eV (700 °C). *p*_*trap*(*DOS*)_ was estimated using these parameters, extracted *N*_*tv*_ (see Fig. 1b), *E*_*u*_ in Table 1 and Equation (11), and *p*_*trap*(*Hall*)_ was calculated using Equation (7) as seen in Fig. 2a. In order to check the extent of the discrepancy between *p*_*trap*(*Hall*)_ and *p*_*trap*(*DOS*)_, we assumed *α*_*GLC*_ = 1 for *T*_*A*_ = 700 °C (i.e. *p*_*trap*(*Hall*)_ (700 °C) = *p*_*trap*_ (700 °C)) based on the fact that a Cu_2_O TFT annealed at 700 °C follows the Meyer-Neldel (MN) rule indicating that carrier transport is governed by trap-limited conduction^13, 22^ (see Supplementary Fig. S1), and *p*_*trap*(*DOS*)_ was corrected by the product of *p*_*trap*(*DOS*)_ (normalized to the value at *T*_*A*_ = 700 °C) and *p*_*trap*(*Hall*)_ (700 °C) (see *p*_*trap*(*corr*)_ in Fig. 2a). Note that *p*_*trap*(*Hall*)_ values for all cases except the material annealed at 700 °C (i.e. *α*_*GLC*_ < 1) are overestimated *p*_*trap*_ values which are simply used for the extraction of *α*_*GLC*_; they are not actual values of *p*_*trap*_. Figure 2a shows a large discrepancy between *p*_*trap*(*Hall*)_ and *p*_*trap*(*corr*)_ for the as-deposited film; the discrepancy decreases significantly after high-temperature annealing. Representing Equation (6) allowing for *α*_*GLC*_ and explicitly including *p*_*trap*(*Hall*)_ and *p*_*trap*(*corr*)_ gives

14 $${p}_{trap(corr)}=[(\frac{{\mu }_{0}}{{\mu }_{Hall}}-1)-\frac{{\mu }_{0}}{{\mu }_{Hall}}(1-{\alpha }_{GLC})]{p}_{free}={p}_{trap(Hall)}-\frac{{p}_{free}{\mu }_{0}}{{\mu }_{Hall}}(1-{\alpha }_{GLC}).$$

*α*_*GLC*_ is then given by

15 $${\alpha }_{GLC}=1-\frac{{\mu }_{Hall}}{{\mu }_{0}}(\frac{{p}_{trap(Hall)}-{p}_{trap(corr)}}{{p}_{free}}).$$

Using Equation (15) and the difference between *p*_*trap*(*Hall*)_ and *p*_*trap*(*corr*)_, *α*_*GLC*_ values as a function of *T*_*A*_ were finally extracted as shown in Fig. 2b. This shows that a very low *α*_*GLC*_ (~0.001) of the as-deposited film increases significantly after annealing at 500 °C and *α*_*GLC*_ approaches unity as *T*_*A*_ increases further. This suggests that GLC significantly affects hole transport in as-deposited Cu_2_O but the effect of GLC on hole transport becomes insignificant after high-temperature annealing.

### Extraction of *μ*_0,*GLC*_ and *μ*_*TLC*_ from *μ*_*Hall*_

*μ*_0,*GLC*_ (the effective carrier mobility reduced by GLC) and *μ*_*TLC*_ were extracted from the measured *μ*_*Hall*_ using the extracted *α*_*GLC*_ and Matthiessen’s rule in order to quantitatively investigate the relative dominance of TLC and GLC. *μ*_*GLC*_ is given using Equation (5) and *μ*_*Hall*_ = *μ*_0_ *α*_*GLC*_ *β*_*TLC*_ (see Equation (4)) as follows,

16 $${\mu }_{GLC}=\frac{{\mu }_{0}{\alpha }_{GLC}{\beta }_{TLC}}{1-{\alpha }_{GLC}}=\frac{{\mu }_{Hall}}{1-{\alpha }_{GLC}}.$$

The assumption (*α*_*GLC*_ = 1 for *T*_*A*_ = 700 °C, i.e. GLC has no effect on carrier mobility) leads to *μ*_*GLC*_ = ∞, which physically means the relative insignificance of GLC compared to TLC (i.e. *μ*_*GLC*_ $\gg$ *μ*_*TLC*_), not actually infinite *μ*_*GLC*_. Because of the calculated infinite value of *μ*_*GLC*_ for *T*_*A*_ = 700 °C, *μ*_0,*GLC*_ was calculated using Matthiessen’s rule (i.e. ${\mu }_{0,{GLC}}^{-1}={\mu }_{0}^{-1}+{\mu }_{{GLC}}^{-1}$) and Equation (16) for a quantitative comparison with *μ*_*TLC*_,

17 $${\mu }_{0,GLC}=\frac{{\mu }_{0}{\mu }_{Hall}}{{\mu }_{0}(1-{\alpha }_{GLC})+{\mu }_{Hall}}.$$

*μ*_*TLC*_ was calculated using the following equation derived from Matthiessen’s rule (i.e. ${\mu }_{{Hall}}^{-1}\,=$ ${\mu }_{0}^{-1}+{\mu }_{GLC}^{-1}+{\mu }_{TLC}^{-1}={\mu }_{0,GLC}^{-1}+{\mu }_{TLC}^{-1}$),

18 $${\mu }_{TLC}=\frac{{\mu }_{0,GLC}{\mu }_{Hall}}{{\mu }_{0,GLC}-{\mu }_{Hall}}.$$

**Figure 3:**
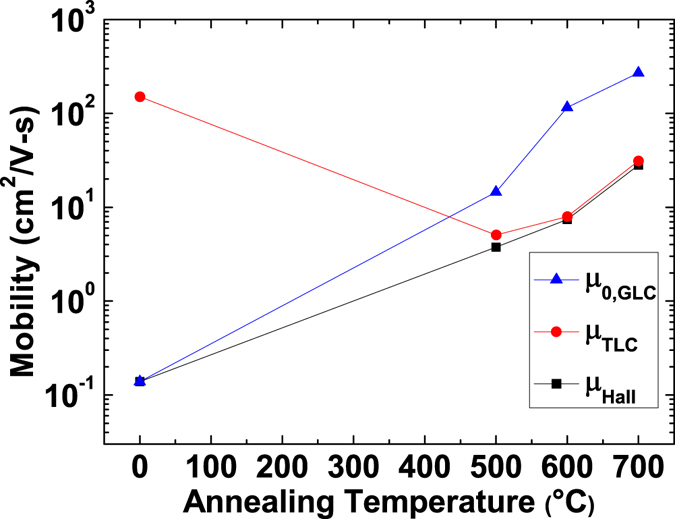
Measured *μ*_*Hall*_, extracted *μ*_*TLC*_ and *μ*_0,*GLC*_ as a function of annealing temperature. *μ*_*Hall*_ is entirely determined by *μ*_0,*GLC*_ for the as-deposited Cu_2_O thin film, whereas *μ*_*Hall*_ is limited by *μ*_*TLC*_ after high-temperature annealing, which suggests that GLC dominates before annealing and TLC becomes dominant after high-temperature annealing.

The calculated results (see Fig. 3) show that *μ*_*Hall*_ of as-deposited Cu_2_O is governed by GLC, suggesting that the very low *μ*_*Hall*_ of the Cu_2_O thin film before annealing is due to the considerable GLC. In contrast, GLC becomes insignificant and therefore TLC dominates after post-deposition annealing at temperatures ≥500 °C.

### Explanation for the change in the effects of GLC & TLC

**Figure 4:**
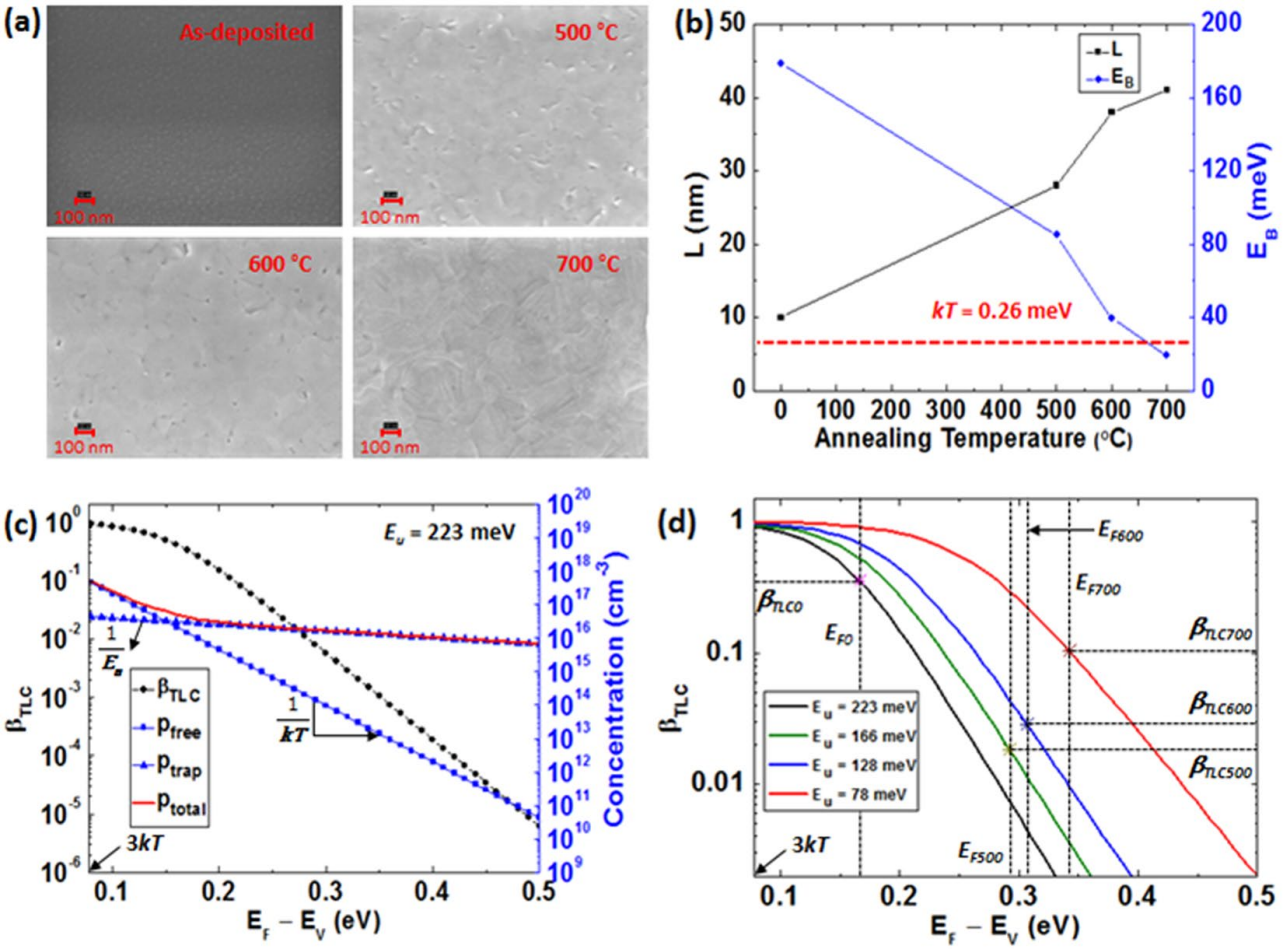
(**a**) SEM images of the surface of as-deposited and annealed Cu_2_O films, (**b**) the estimated grain size (*L*) and energy barrier height at grain boundaries (*E*_*B*_) as a function of annealing temperature, (**c**) calculated *p*_*free*_, *p*_*trap*_, *p*_*total*_ and *β*_*TLC*_ when *E*_*u*_ = 223 meV and (d) *β*_*TLC*_ when *E*_*u*_ = 223 meV (as-deposited), *E*_*u*_ = 166 meV (500 °C), *E*_*u*_ = 128 meV (600 °C) and *E*_*u*_ = 78 meV (700 °C) as a function of *E*_*F*_ − *E*_*V*_. In (**b**), the red dot line shows the thermal energy at room temperature. In (**c**), *p*_*free*_ was calculated using Equation (12) given by Boltzmann’s approximation which is valid for *E*_*F*_ − *E*_*V*_ ≥ 3 *kT*. In (**d**), *E*_*F*0_, *E*_*F*500_, *E*_*F*600_ and *E*_*F*700_ denote the Fermi energy of the as-deposited film and 500, 600 and 700 °C-annealed films, and the corresponding *β*_*TLC*_ values are *β*_*TLC*0_ = 0.36, *β*_*TLC*500_ = 0.018, *β*_*TLC*600_ = 0.029 and *β*_*TLC*700_ = 0.1, respectively.

To explain the reduction in the GLC effect with an increase in *T*_*A*_, changes in the grain size (*L*) and the energy barrier height at the grain boundary (*E*_*B*_) were examined. SEM images (see Fig. 4a) show that *L* tends to increase with increasing *T*_*A*_, but it is hard to quantitatively provide *L* values since annealed thin films have irregular grains. In order to examine the change in *L* quantitatively, *L* was extracted from the Scherrer equation^23^ (i.e. *L* = 0.94*λ*/(*β* cos *θ*)) using the line broadening of the intense Cu_2_O (200) peak of the XRD patterns reported in the authors’ previous paper^8^. Here, *λ* and *θ* are the X-ray wavelength of Cu K_α1_ radiation (0.154 nm) and the Bragg angle, respectively, and *β* denotes the full width at half maximum (FWHM) corrected by ${({\beta }_{m}^{2}-{\beta }_{i}^{2})}^{1/2}$ where *β*_*m*_ and *β*_*i*_ are the measured and instrumental FWHM in radians. The grain sizes from the SEM images (see Fig. 4a) and extracted from the Scherrer equation (see Fig. 4b) do not match exactly. This can be understood by considering that the grain size observed from the SEM images can be different from the extracted value since the SEM images show a small surface area while XRD samples the area of the X-ray beam size (the beam diameter: ~500 μm). The effective mobility by GLC is given as ${\mu }_{0,GLC}=Lq\sqrt{1/2\pi {m}^{\ast }kT}\exp (-{E}_{B}/kT)$, where *q* is the elementary charge^5^. Using this equation, the *E*_*B*_ is expressed as

19 $${E}_{B}=-kT\,\mathrm{ln}\,(\frac{{\mu }_{0,GLC}\sqrt{2\pi {m}^{\ast }kT}}{Lq}).$$

*E*_*B*_ was calculated using the extracted values (i.e. *L* and *μ*_0,*GLC*_) and Equation (19). The calculated results (see Fig. 4b) quantitatively show that an increase in *T*_*A*_ leads to an increase in *L* and a decrease in *E*_*B*_, which provides a clear explanation for the reduction in the GLC effect. Additionally, *E*_*B*_ decreases to *E*_*B*_ < *kT* (i.e. ~26 meV at 300 K) at *T*_*A*_ = 700 °C, which is considered to be the main reason for the insignificance of GLC in the 700 °C-annealed Cu_2_O thin film. In addition, based on Equations (2) and (3), the change in *μ*_*TLC*_ can be explained by the variation in *β*_*TLC*_ (i.e. the ratio of *p*_*free*_ to *p*_*free*_ + *p*_*trap*_). Figure 4c shows calculated *p*_*free*_, *p*_*trap*_ = *p*_*trap*(*corr*)_, *p*_*total*_ = *p*_*free*_ + *p*_*trap*_ and *β*_*TLC*_ when *E*_*u*_ = 223 meV (extracted from the as-deposited case) as a function of *E*_*F*_ − *E*_*V*_. This clearly shows that *μ*_*TLC*_ is affected by not only *E*_*u*_ but also the position of *E*_*F*_. Specifically, as *E*_*F*_ − *E*_*V*_ decreases, *p*_*free*_ increases more significantly than *p*_*trap*_ due to (*kT*)^−1^ > (*E*_*u*_)^−1^ and therefore *β*_*TLC*_ approaches unity as seen in Fig. 4c. This means a reduction in the TLC effect as *E*_*F*_ approaches to *E*_*V*_. Figure 4d shows the calculated *β*_*TLC*_ of all the samples (i.e. when *E*_*u*_ = 223 meV (as-deposited), *E*_*u*_ = 166 meV (500 °C), *E*_*u*_ = 128 meV (600 °C) and *E*_*u*_ = 78 meV (700 °C)) as a function of *E*_*F*_ − *E*_*V*_. If all the samples had the same *E*_*F*_, the as-deposited film with the highest *E*_*u*_ would lead to the lowest *β*_*TLC*_. However, since *E*_*F*_ − *E*_*V*_ of the as-deposited film is much smaller compared to annealed films, its *β*_*TLC*_ is the highest, as seen in Fig. 4d, which is the reason for the relatively insignificant TLC effect in the as-deposited film.

### The density of copper vacancies $({{\boldsymbol{N}}}_{{{\boldsymbol{V}}}_{{\boldsymbol{Cu}}}})$

As seen in Table 1 and Fig. 2a, an increase in *T*_*A*_ leads to a reduction in the total hole concentration (*p*_*free*_ + *p*_*trap*_), suggesting a decrease in *V*_*Cu*_ which is the main hole producer in Cu_2_O. In order to provide a quantitative insight into the reduction in *V*_*Cu*_ with an increase in *T*_*A*_, the density of copper vacancies, ${{N}}_{{{V}}_{{Cu}}}$, was extracted using the following method. This method begins with the charge neutrality condition, ${p}+{{N}}_{{D}}^{+}={n}+{{N}}_{{A}}^{-}$, where *p*, *n*, ${N}_{D}^{+}$ and ${N}_{A}^{-}$ are the densities of free holes, free electrons, ionized donors and ionized acceptors, respectively. Since Cu_2_O films have valence band tail states (i.e. donor-like states) and holes are trapped at the tail states, the density of ionized tail states (${N}_{TS}^{+}$) should be added into the charge neutrality condition (i.e. $p+{N}_{D}^{+}+{N}_{TS}^{+}=n+{N}_{A}^{-}$). This becomes $p+{N}_{TS}^{+}$ ≈ ${N}_{A}^{-}$ by considering ${N}_{D}^{+}$ and *n* to be negligible (i.e. $p+{N}_{TS}^{+}\gg {N}_{D}^{+}$, ${N}_{A}^{-}\gg n$). In addition, since another possible hole producer (the oxygen interstitial, *O*_*i*_) has deep acceptor levels, it can be assumed that *V*_*Cu*_ with the shallow acceptor level dominates the generation of holes in Cu_2_O^1^. Thus, ${N}_{A}^{-}$ can be substituted with the density of ionized *V*_*Cu*_ $({N}_{{V}_{Cu}}^{-})$. Finally, because ${N}_{TS}^{+}={p}_{trap}$, we can obtain the following equation,

20 $${N}_{{V}_{Cu}}^{-}\approx {p}_{free}+{p}_{trap}.$$

Using the formula describing the fraction of ionized acceptor concentration^21^, ${N}_{{V}_{Cu}}^{-}$ could be given as follows,

21 $${N}_{{V}_{Cu}}^{-}=\frac{{N}_{{V}_{Cu}}}{1+{g}_{A}\,\exp \,(\frac{{E}_{{V}_{Cu}}-{E}_{F}}{kT})},$$

where *g*_*A*_ and ${E}_{{V}_{Cu}}$ are the acceptor-site degeneracy factor and the energy level of copper vacancies, respectively. Here, representing ${E}_{{V}_{Cu}}-{E}_{F}$ as $({E}_{{V}_{Cu}}-{E}_{V})+({E}_{V}-{E}_{F})$, Equation (21) can be expressed as

22 $${N}_{{V}_{Cu}}^{-}=\frac{{N}_{{V}_{Cu}}}{1+{g}_{A}\,\exp \,(\frac{{E}_{{V}_{Cu}}-{E}_{V}}{kT})\,\exp \,(\frac{{E}_{V}-{E}_{F}}{kT})},$$

Using Equations (12), (20) and (22), ${N}_{{V}_{Cu}}$ is expressed as

23 $${N}_{{V}_{Cu}}=\,({p}_{free}+{p}_{trap})[1+{g}_{A}\,\exp \,(\frac{{E}_{{V}_{Cu}}-{E}_{V}}{kT})\frac{{p}_{free}}{{N}_{V}}].$$

**Figure 5:**
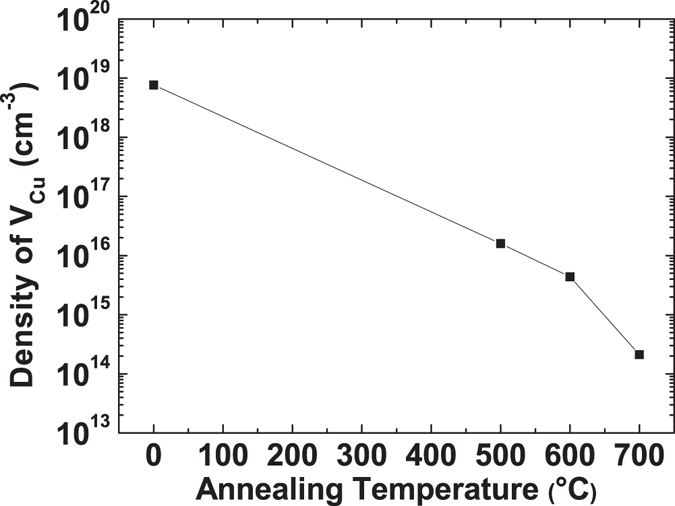
Extracted density of copper vacancies as a function of annealing temperature.

Here, ${E}_{{V}_{Cu}}-{E}_{V}$ = 0.28 eV is used; this has been calculated by density-functional theory (DFT)^1^. Since Cu_2_O has one degenerate valence band (${{\rm{\Gamma }}}_{7}^{+}$) at the VBM^18^, each copper vacancy state (i.e. each acceptor state) can accept one hole with either spin or have no hole^21^, and hence *g*_*A*_ = 2. Using these parameters (i.e. the measured *p*_*free*_, calculated *p*_*trap*(*corr*)_ for *p*_*trap*_ and Equation (23)), ${N}_{{V}_{Cu}}$ was extracted as seen in Fig. 5, which quantitatively shows a significant decrease in *V*_*Cu*_ with an increase in *T*_*A*_.

## Conclusions

In conclusion, this paper shows that grain-boundary-limited conduction becomes insignificant and carrier transport is governed by trap-limited conduction after high-temperature annealing. This is explained by a considerable reduction in the energy barrier height at the grain boundaries and an increase in the grain size by high-temperature annealing, suggesting that the GLC effect on hole transport in Cu_2_O can be reduced significantly by post-deposition annealing. In addition, an increase in annealing temperature gives rise to a decrease in the total hole concentration, suggesting a reduction in copper vacancies, which is the main origin of holes in Cu_2_O. An extraction method for the density of copper vacancies $({N}_{{V}_{Cu}})$ is proposed and the consequent calculation of ${N}_{{V}_{Cu}}$ quantitatively shows a significant decrease in copper vacancy density with annealing.

## Methods

### Film Fabrication

Deposition of Cu_2_O was performed by remote-plasma reactive sputtering using a high target utilization sputtering (HiTUS) system (Plasma Quest Limited) without intentional substrate heating (see a schematic diagram of HiTUS^24^). The chamber was pumped to a base pressure of 6.0 × 10^−6^ mbar and Ar gas was supplied to set a process pressure of 1.5 × 10^−3^ mbar. Ar plasma was generated by an RF launch power of 1.2 kW in a remote chamber and then directed onto a metallic copper target with 4 inch diameter and 99.999% purity by electromagnets in the chamber. The reactive sputtering was performed at an oxygen flow rate of 16 sccm and a DC bias power of 0.95 kW with a DC bias voltage of ~690 V.

Cu_2_O samples with film thickness of ~500 nm were deposited on quartz (Spectrosil B) and 8 mm × 8 mm glass (Corning 7059) substrates. The thickness was determined using surface profilometry (Veeco Dektak 200SI). The as-deposited Cu_2_O was subsequently annealed in vacuum (9.5 × 10^−4^ mbar) in an Aixtron Cambridge Nanoinstruments Black Magic 2 system at various temperatures (500, 600 and 700 °C) for 10 min. The temperature ramp rate, cooling time and unloading temperature were 5 °C/s, 20 min and 50 °C, respectively. Annealing temperature was monitored with an infrared (IR) radiation pyrometer (Infratherm IGA8 plus). In order to perform Hall measurements using the van der Pauw method, four Au electrodes were thermally evaporated at the corners of the Cu_2_O film deposited on 8 mm × 8 mm glass substrates through a shadow mask.

### Measurement

In order to obtain electrical characteristics (i.e. Hall mobility and carrier density) of Cu_2_O films, Hall measurements at room temperature were carried out using an MMR Technologies Hall Effect Measurement System (K2500-7). For extraction of the Urbach energy (*E*_*u*_), the optical absorption coefficient (α(*υ*)) was obtained using an ATI Unicam UV/Vis spectrometer (UV2-200) and Cu_2_O films formed on quartz substrates. Based on the relation between α(*υ*) and *E*_*u*_, α(*υ*) = α_0_ exp(*hυ*/*E*_*u*_), *E*_*u*_ was extracted from the reciprocal of the slopes of the linear region of an ln(α) versus *hυ* plot^8^.

### Data Availability

The datasets generated during and/or analysed during the current study are available in the Cambridge University Data Repository (http://www.repository.cam.ac.uk/).

## Electronic supplementary material

Supplementary Information
