# Supplementary material for: Analysis of the Conduction Mechanism and Copper Vacancy Density in p-type Cu2O Thin Films
Source: Sci Rep. 2017 Jul 18;7:5766. doi: 10.1038/s41598-017-05893-x (PMC5515854; doi:10.1038/s41598-017-05893-x)
Supplement: Supplementary file 1 — Supplementary Information [file 41598_2017_5893_MOESM1_ESM.pdf]

Supplementary Information

Analysis of the Conduction Mechanism and Copper Vacancy Density in p-type Cu_2O Thin Films

Sanggil Han & Andrew J. Flewitt*

Electrical Engineering Division, Department of Engineering, University of Cambridge, Cambridge CB3 0FA, United Kingdom

*Corresponding author: e-mail (ajf@eng.cam.ac.uk)

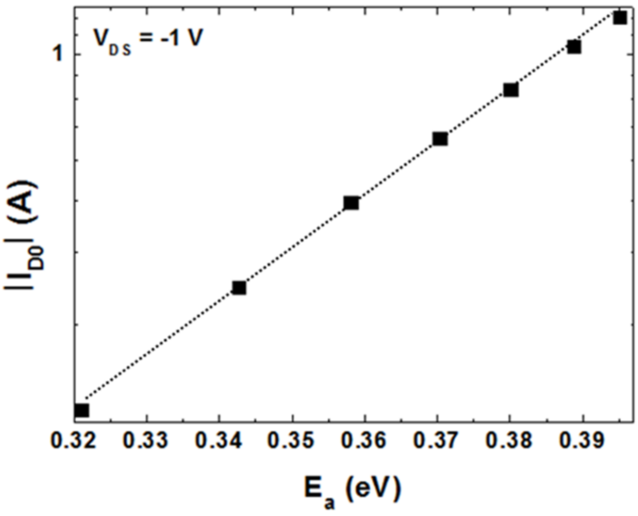

Figure S1. Prefactor ($|I_{D0}|$) (log scale) versus activation energy (E_a). I_{D0} and E_a were extracted from $I_D = I_{D0}\exp(-E_a/kT)$[12]. I_D is measured drain current of a Cu_2O TFT annealed at 700 ℃ according to variation of gate voltage and measurement temperature at drain voltage of -1 V. The Meyer-Neldel (MN) rule is an exponential relation between I_{D0} and its E_a, and the appearance of the MN rule represents trap-limited conduction[12,19].
